# Medical News and Items

**Published:** 1878-06

**Authors:** 


### Medical News and Items.

---

THE PHONOGRAPH opens up a vista of medical possibilities delightful to contemplate. Who can fail to make the nice distinctions between every form of bronchial and pulmonary râle, percussion, succussion and friction sounds, surgical crepitus, fœtal and placental murmurs, and arterial and aneurismal bruit, when each can be produced at will, amplified to any desired extent, in the study, the amphitheater, the office and the hospital! The lecturer of the future will teach more effectually with this instrument than by the mouth. The phonograph will record the frequency and characteristics of respiratory and muscular movements, decide as to the age and sex of the fœtus in utero, and differentiate pneumonia from phthisis. It will reproduce the sob of hysteria, the sigh of melancholia, the singultus of collapse, the cry of the puerperal woman in the different stages of labor. It will interpret for the speechless infant, the moans and cries of tubercular meningitis, ear-ache and intestinal colic. It will furnish the ring of whooping-cough and the hack of the consumptive. It will be an expert in insanity, distinguishing between the laugh of the maniac and the drivel of the idiot. It will classify dysphasic derangements, such as ataxic, amnesic, paraphasic and akataphasic aphasia.

It will recount, in the voice and words of the patient, the agonies of neuralgia and renal calculus, and the horrors of delirium tremens. It will give the burden of the story of the old lady who recounts all the ills of her ancestors before proceeding to the era of her own. More than this, it will accomplish this feat in the ante-room, while the physician is supposed to be busying himself with his last patient.

Last but not least, it will simultaneously furnish to the medical philosopher, the grateful praises and promises of him who is convalescent from dangerous illness, together with the chilling accents in which, later, the doctor is told that he must wait for his remuneration till the butcher and the baker have been paid.

THE third annual session of the *Arkansas State Medical Society* convened in Adelaide Hall, in the city of Fort Smith, Wednesday morning, May 1st, 1878.

Dr. A. N. Carrigan, President, delivered the annual address. He congratulated the Society upon the favorable auspices that surrounded them at this meeting; spoke of its standing before the American Medical Association; referred to the late struggle before this body, of the success it had met with, and the good that had resulted to medical science throughout the State, by the interest awakened through the agitation caused by the rival effort for recognition.

He spoke of malarial influences throughout the State that would probably be relieved by proper hygienic measures, and for this object recommended the establishment of a Board of Health by the General Assembly of the State.

The session lasted two days, and a great number of interesting essays were read.

The officers for the ensuing year are: President—A. A. Horner, of Phillips County. Vice-Presidents—T. W. Hurley, of Benton County; W. H. Hawkins, of Little River County; J. S. Shibbley, of Logan County; Isaac Folsom, of Lonoke County. Secretary—R. G. Jennings. Assistant Secretary—L. P. Gibson. Treasurer—A. L. Breysacher. Librarian—J. H. Lenow.

Next place of meeting, Little Rock, the first Wednesday in May, 1879.

THE rise and progress of what is called the science of therapeutics is merely, I believe, a modern development of professional credulity.—W. LANDER LINDSAY, M. D., F. R. S. E., *Medical Times and Gazette, March* 16, 1878.

THE Alumni Association of the College of Physicians and Surgeons in the city of New York, offer for the following year a prize of ($500) five hundred dollars, open for competition to all alumni of the college. It will be awarded to the best medical essay submitted, if deemed sufficiently meritorious, upon any subject which the writer may select. The essay, in order to compete, must be based upon *original investigation.* Each essay must be marked with a device or motto, and accompanied by a sealed envelope, similarly marked, containing the name and address of the author. Essays must be submitted to the Prize Committee on or before Feb. 15th, 1879. They may be sent directly to any of the committee, at the college, care of the Secretary. The committee consists of Drs. Henry B. Sands, Wm. H. Draper and Frank E. Beckwith.

ONE thousand four hundred married women accompanied 10,827 British soldiers to the Madras Presidency in India, and produced 2,900 children, now living, not one of whom has had syphilis. This speaks favorably for the English plan of allowing a certain number of wives to accompany each regiment; as nearly 20 per cent. of the Indian troops suffer from venereal disease.

THE thirteenth annual report of the Chicago hospital for women and children, which has just made its appearance, is a gratifying exhibit of the work accomplished by this deserving charity. We observe eight typographical errors in the physician's report that occupies but little more than one page, and could wish that these had been corrected in a feminine postscript.

THIS is the last number of Volume XXXVI. The index will be mailed with the first number (July) of the next volume.

If any of our patrons, whose time expires this month, do not wish to continue their subscription, they may obtain the index by notifying us.

WE have to express our obligations to Prof. N. S. Davis of Chicago, for the report prepared by him and published in this number, of the proceedings of the Illinois State Medical Society.

